# Unusual Presentation of a Rare Pneumothorax in a Patient With COVID-19 Pneumonia: A Case Report

**DOI:** 10.7759/cureus.19273

**Published:** 2021-11-05

**Authors:** Mac Josh Reandelar, Elizabeth Park, Celestina D'Costa, Armish Salahudin, Yusufal Mamoon

**Affiliations:** 1 Medicine, Icahn School of Medicine at Mount Sinai, Queens Hospital Center, Jamaica, USA

**Keywords:** cytokine storm syndrome, sars-cov-2 (severe acute respiratory syndrome coronavirus -2), covid-19 pneumonia, covid 19, pneumothorax (ptx)

## Abstract

Coronavirus disease 2019 (COVID-19) is a respiratory and systemic disease caused by severe acute respiratory syndrome coronavirus 2 (SARS-CoV-2) infection. Since the start of the COVID-19 pandemic, pneumothorax (PTX) has only been reported as a complication of the virus-induced pneumonia in less than 1% of cases. The majority of them developed symptoms in the setting of either an underlying history of lung disease or being placed on a mechanical ventilator during admission. The authors report a unique case of PTX in a patient with a recent COVID pneumonia that did not fit the aforementioned clinical picture - a 41-year-old male with a complete collapse of his right lung who was previously admitted for COVID pneumonia with no known pulmonary history and was not intubated. A chest tube was placed with the resolution of the PTX and the patient is being monitored on the medicine floor.

## Introduction

Since the World Health Organization (WHO) published their first disease outbreak news on what was then a “new virus” in the January of 2020, our understanding of the coronavirus disease 2019 (COVID-19) has changed drastically. As numbers across the globe continue to waver, so does our grasp on the extent of this disease. Reports from within the last year have detailed many of its associated complications, from myocarditis [1], to neurological dysfunctions [2], new-onset type I diabetes [3], and gastrointestinal bleeding [4]. Recognizing these complications has helped medical teams establish and continuously modify protocols for inpatient and outpatient management of COVID patients.

In this article, we present a new case of pneumothorax (PTX) in a 41-year-old male who was previously admitted for COVID-19 pneumonia. The patient was discharged from the first admission on home supplemental oxygen and reported significant improvement. More than a week later, he returned to the emergency room with complaints of worsening dyspnea. The patient was found to have a large right-sided PTX with a significant left mediastinal shift. A chest tube was placed and he was transferred to the intensive care unit (ICU) for further management of unstable laboratory results. Once stabilized, he was transferred to the medicine floor for monitoring PTX resolution. Of significant interest, this patient was not mechanically ventilated during his admission for COVID pneumonia and had no known history of underlying lung disease prior. His disease course is notably different from those who have been previously reported by authors exploring the association between COVID-19 infection and pneumothoraces.

## Case presentation

Informed consent was obtained from the patient himself. A 41-year-old obese male, 23-pack a year former smoker with poor primary care follow up and a past medical history of uncontrolled hypertension (HTN) and uncontrolled type II diabetes mellitus (DM) presented to the emergency department with worsening dyspnea. This patient was admitted 20 days before for sepsis and acute hypoxic respiratory failure in the setting of COVID pneumonia without requiring intubation. The patient was not vaccinated against COVID-19 and had no history of lung disease prior. During the previous admission, the patient had bilateral rales on examination and was saturating at 91%-92% on room air with improvement to 3% on 3 L nasal cannula. His laboratory results at that time were significant for neutrophilic leukocytosis, borderline anemia, and significantly elevated inflammatory markers [D-dimer, C-reactive protein (CRP), IL-6, lactate dehydrogenase (LDH), and ferritin] as seen in Table 1.

**Table 1:** Pertinent lab findings on the day of the patient’s first admission for COVID pneumonia. Labs were significant for leukocytosis, anemia, and abnormal inflammatory markers WBC, white blood cell; RBC, red blood cell; HGB, hemoglobin; HCT, hematocrit; MCV, mean corpuscular volume; LDH, lactate dehydrogenase; CRP, C-reactive protein

Labs	Reference range	Value
WBC	4.80 - 10.80 x 10(3)/mcL	14.53
RBC	4.70 - 6.10 x 10(6)/mcL	4.56
HGB	14.0 - 18.0 g/dL	14.1
HCT	42.0% - 52.0%	42.5
MCV	80.0 - 99.0 fL	93.2
Neutrophil %	44.0% - 70.0%	89.1
Lymphocyte %	20.0% - 45.0%	6.5
Monocyte %	2.0% - 10.0%	3.6
Eosinophil %	1.0 - 4.0 %	0.0
Basophil %	0.0 - 2.0 %	0.1
Imm Gran %	0.0 - 2.0 %	0.7
D-Dimer Quant DDU	0 - 243 ng/mL	1,211
High sensitive CRP	<=5.00 mg/L	162.50
Interleukin-6	0.0 - 13.0 pg/mL	12.5
LDH	135 - 225 U/L	1602
Ferritin	30 - 400 ng/mL	6043

**Figure 1:**
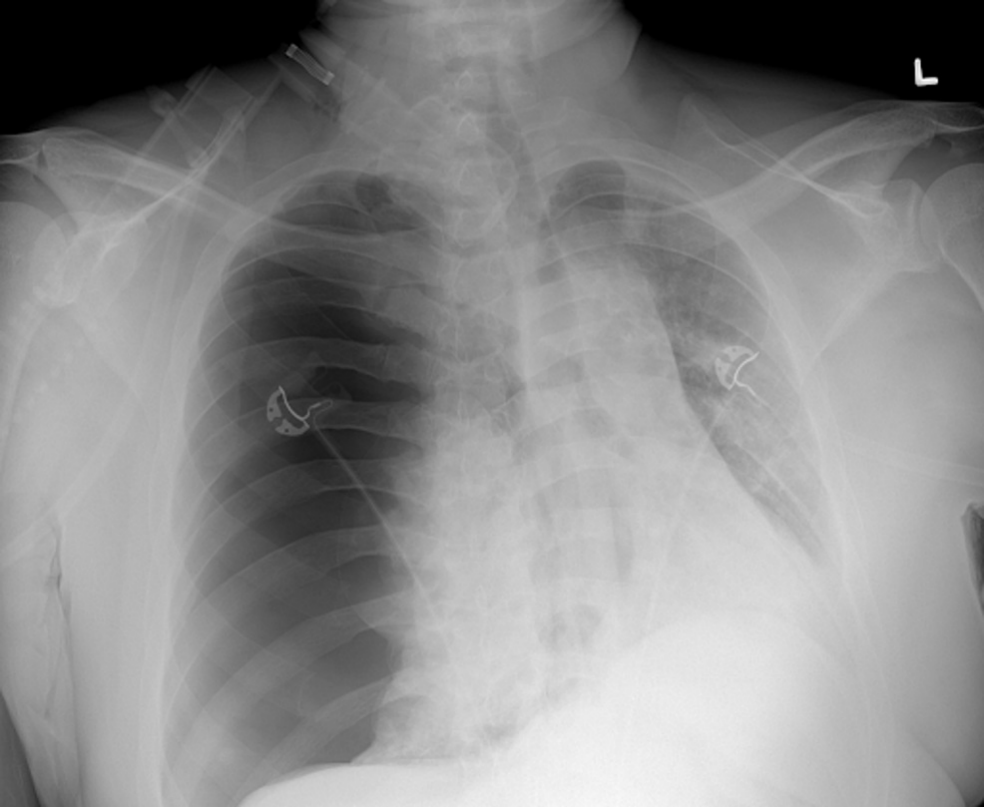
Chest X-ray from the ED upon presentation. Large right sided pneumothorax with complete collapse of the right lung. Shift of mediastinum and heart to left consistent with tension pneumothorax. Proximal density left lung which may be related to tension pneumothorax or residual consolidation which was noted on patient's prior chest X-ray. ED, emergency department

**Figure 2:**
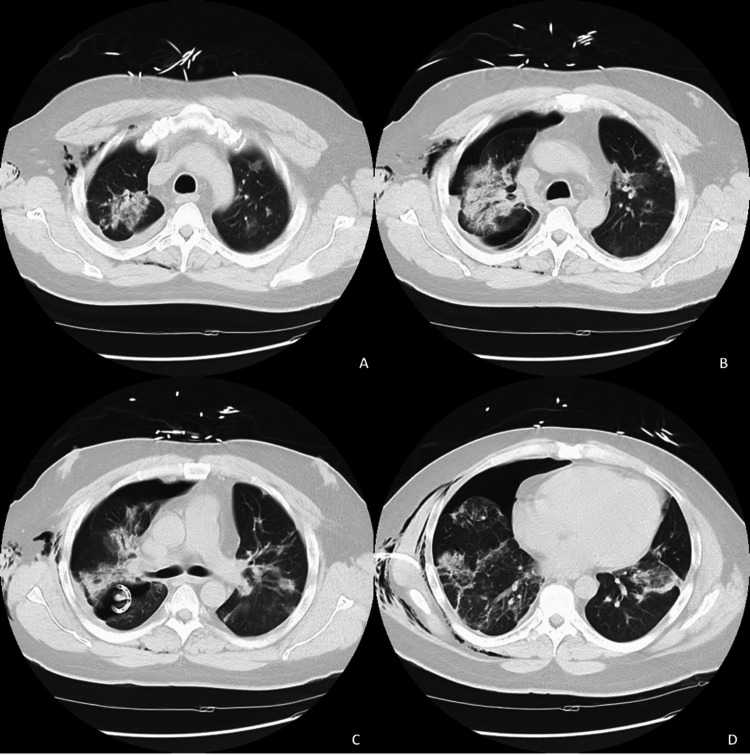
Diagnostic CT of the chest without contrast. Right chest catheter terminates in the posterior aspect of the major fissure. There is a moderate to large-sized right pneumothorax, small right pleural fluid collection, and extensive bilateral patchy pulmonary opacities appear more consolidated.

**Figure 3:**
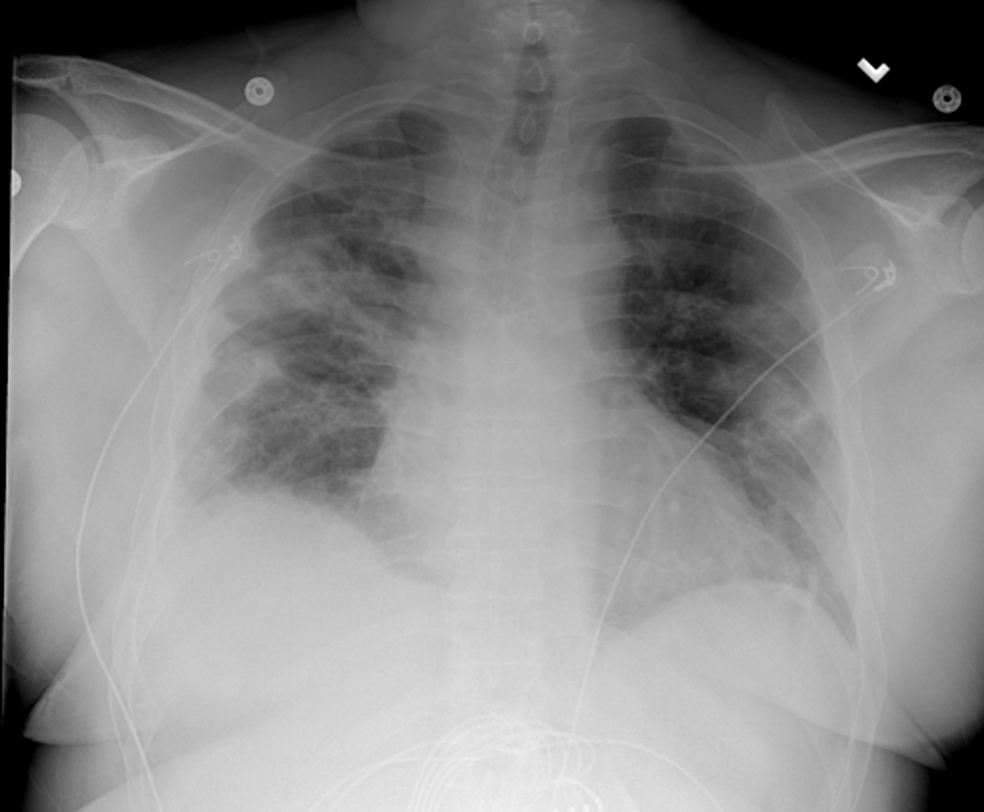
Chest radiograph status post pigtail removal. Image taken one day before discharge. Previously placed pigtail catheter in the right hemithorax has been removed. Trachea is midline. Cardiac silhouette is grossly unchanged. Patchy opacities in the left mid and lower lung field are unchanged from prior. No gross pneumothorax. Alveolar opacities in the right mid and lower lung fields appear stable to minimally improved. Suspect small right pleural effusion. Visualized osseous structures are grossly unremarkable.

The patient was started on remdesivir and prednisone 10 mg for five days while on 4L Oxymask. He was then discharged six days later with supplemental oxygen as needed. The patient felt well at the time of discharge and had no complaints for about one week. He then had some difficulty breathing 8-10 days since his discharge. On the 11th day, his symptoms significantly worsened despite oxygen supplementation, prompting his return to the emergency room. As per the electronic medical record (EMR), the patient was not hypotensive but was tachypneic at 111 beats per minute upon arrival, only speaking few-word sentences. He was in obvious respiratory distress with absent breath sounds on the right side, but his oxygen saturation was stable at 98%-100% on a non-rebreather mask. A chest X-ray (Figure 1) revealed a “large right PTX with a complete collapse of the lung” and “shift of mediastinum and heart to left consistent with tension PTX” so a pigtail catheter was placed with significant improvement of PTX during insertion. A CT of the chest without contrast was subsequently ordered, which confirmed the resolving PTX and revealed bilateral patchy pulmonary opacities as well as a small right pleural fluid collection, likely residual from his recent COVID pneumonia (Figure 2A-D). Due to poor primary care follow-up, no prior imaging studies were available to definitively rule out any underlying history of emphysematous disease. The patient was started on cefepime and vancomycin for empiric coverage. The patient tolerated the procedure well and was followed by the surgical team during his entire length of stay. Daily chest X-rays showed progressive resolution of the PTX (Figure 3), the pain was well controlled, and he denied any further respiratory distress. The patient’s vital signs remained stable for the rest of his admission, comfortably saturating within the 95%-99% range on room air. On the 12th day of admission, the pigtail catheter was removed. The patient was breathing comfortably and saturating well on room air at 95%-96%, and was discharged with outpatient surgery follow-up.

## Discussion

Recent studies have presented PTX as a rare (seen in less than 1% of cases) complication of COVID pneumonia [5-7]. However, this should raise concern because COVID-19-related pneumothoraces are associated with prolonged hospitalizations, increased likelihood of ICU admission, and death, especially among the elderly [8]. According to other case reports, patients who presented with this complication either had an underlying lung disease were mechanically ventilated during the hospital course or developed the PTX within the timeline of the same admission [9]. Our patient had no known underlying lung pathology (albeit a former chronic smoker), did not require intubation, and was stabilized for discharge from his COVID admission before developing the PTX more than one week later, making his case a unique point of interest.

Pneumothorax develops when air enters the pleural space as a result of disease or injury. The resulting partial or complete collapse of the lung parenchyma is due to a loss of negative pressure between the visceral and parietal pleural membranes. The two main classifications are spontaneous and traumatic, both of which can progress to tension PTX and can lead to life-threatening complications. Many proposed mechanisms explain the relationship between COVID-19 infection and the development of a PTX. These include inflammation from the “cytokine storm,” parenchymal injury, ischemia, infarction, cough, or a pneumatocele rupture [10]. 

A frequently cited cause is barotrauma mostly seen in patients with acute respiratory distress syndrome (ARDS) who were placed on mechanical ventilation [11]. This phenomenon was also reported by Zantah et al. in a retrospective study where four of the six COVID-19 patients who developed a PTX were intubated [9]. Our patient, however, did not require intubation, bilevel positive airway pressure (BiPAP), or continuous positive airway pressure (C-PAP) airway assistance which made his chances of getting a PTX less likely. Similarly, increased intrathoracic pressure in the setting of heavy coughing can also lead to the development of barotrauma complications but he did not report any significant bouts of coughing during his hospital stay [12]. 

With regard to inflammation, poorer outcomes in COVID-19 infection are associated with clinical and laboratory findings of cytokine storm syndrome (CSS), which is characterized by hyperinflammation and multiorgan disease [13]. The virus binds to human angiotensin-converting enzyme 2 (ACE2) receptors on host cells, leading to the release of cytokines [14]. A genetic predisposition for an increased number and increased sensitivity to ACE receptors could have caused a higher amount of viral infiltration and in turn, caused extensive inflammatory damage leading to the respiratory collapse. Many patients with CSS will present with blood-count abnormalities such as leukocytosis, leukopenia, anemia, thrombocytopenia, and elevated ferritin and D-dimer levels. Additionally, serum inflammatory cytokine levels such as interferon-𝞬, interleukin-6, interleukin-10, and soluble interleukin-2 receptor alpha are usually elevated [15]. These are consistent with some of the lab findings we saw with our patient during his first admission (Table 1). Of note, some studies show a direct and strong relationship between CRP and severity of disease [16], whereas one study showed that higher interleukin-6 levels are strongly associated with shorter survival [17]. Additionally, a higher expression of a transmembrane serine protease 2 encoded by TMPRSS2 gene disproportionately found in nasal epithelial cells of individuals that self-identified as Black Americans allows for a greater burden of COVID-19 viral entry and spread via airway [18]. We, therefore, assume that, although the patient did not endorse a history of pulmonary disease, the inflammatory response to the virus resulted in enough alveolar damage to cause air to leak through the alveoli and escape into the pleural space [19-20]. These changes likely predisposed him to develop the PTX. Though we are not completely sure of the patient’s medical background given his lack of follow-up, he could have had previously undiagnosed conditions that contributed to his overall clinical picture. 

Given the paucity in reported cases of PTX in COVID-19 patients, our understanding of the predisposing factors for developing this complication is still limited and will require further exploration.

## Conclusions

The late onset of this patient's overlying PTX as well as the lack of characteristic barotrauma and significant pulmonary history lend themselves to the importance of considering every possible complication when caring for patients with COVID-19. As the management of this infection continues to evolve, so should the astuteness to recognize and prevent the course of pulmonary sequelae. 
